# Potential Infectious Complications in Pig Xenograft Donors and Recipients

**DOI:** 10.3389/ti.2024.13594

**Published:** 2025-01-15

**Authors:** Nicolas J. Mueller, Linda Scobie

**Affiliations:** ^1^ Division of Infectious Diseases and Hospital Epidemiology, University Zurich, University Hospital Zurich, Zurich, Switzerland; ^2^ Department of Biological and Biomedical Sciences, School of Health and Life Sciences, Glasgow Caledonian University, Glasgow, Scotland, United Kingdom

**Keywords:** xenotransplantation, preclinical trials, clinical trials, porcine viruses, non-human primates

## Abstract

Preclinical and clinical xenotransplantation trials have shown that successful outcomes depend on a number of factors including the prevention of xenozoonoses. Preclinical trials involving pig kidneys and hearts transplanted into various non-human primates have revealed the potential impact of pig pathogens being present in the transplanted organ/tissue, mainly viruses. The concept of “designated pathogen-free donor animals” was developed to ensure elimination of pathogens during the breeding of donor animals to mitigate this occurrence. This is a challenging process as confirmation of presence and absence of some pathogen, in particular for latent viruses, requires a validated armamentarium of direct and indirect tests. The importance of using the correct diagnostic regimen was highlighted during the first pig-to-man cardiac transplantation with both porcine cytomegalovirus (PCMV), also known as porcine roseolovirus (PRV), and porcine circovirus (PCV) detected in the transplanted organ and in the patient. To further improve xenotransplantation and to achieve trials in Europe it is important that we use these data to inform process for diagnostics both in donor and recipients before and after xenotransplantation to ensure safety. As part of this sensitive and specific pathogen detection systems should be validated and readily available.

## Introduction

Xenotransplantation poses a unique risk of transmitting pathogens from donor animal grafts to human recipients [1]. The main concern is to fully identify any pathogen risk in the donor animal and to then apply appropriate testing mechanisms to ensure that the recipient is at low risk. Indeed, to date, no formal risk analysis framework has been applied.

Specialized breeding techniques and surveillance monitoring can eliminate most potential pathogens from donor animals, particularly bacterial and fungal pathogens, which are usually acquired after birth [2]. However, certain viruses, particularly porcine endogenous retroviruses (PERVs) and latent viruses, present significant challenges. PERVs, with a genomic origin, are passed to offspring and are difficult to inactivate [3]. There have been numerous studies characterizing PERV in many potential donor herds and all aspects related to PERV are summarized in [4].

For many specific xenozoonotic viruses such as Hepatitis E (HEV), accredited and commercially available serological and molecular tests are available, but this is not always the case.

Latent viruses, which are avoidable by early weaning (raising piglets without maternal contact) and caesarean derivation, are hard to detect even with sensitive PCR methods due to ongoing latency. The tissue used for testing is not always suitable for detection in this instance.

With this in mind, this article focuses on viral pathogens in xenotransplantation, what we know about their potential impact on the donor organ/tissue and the recipient and what recommendations can be advised as these are the most challenging for diagnosis and elimination.

## Xenozootic Viral Pathogens and Diagnostics

Standard surveillance and monitoring is mainly carried out by veterinary diagnostic laboratories, e.g., Animal Plant and Health Agency (APHA, United Kingdom). It is always asked, what pathogens do we need to exclude, is there a list?

Such a list can be separated into pathogens that are important for pig health and those that might affect human recipients. It is also important to note that this “list” will be dynamic depending on the location of the xenotransplant donor herd, as emerging and endemic viruses can vary according to geographical site. It is advised that this is a role of the sponsor to generate a suitable surveillance strategy covering all relevant pathogens for the region with input from the relevant veterinary authority. Such a strategy must be in place for any donor herd and checkpoints for testing and a risk analysis available as a standard procedure. It is also important to consider the type of diagnostic method that is being used for the surveillance as serological and molecular assays will convey different information.

Recently, a suggested microbiological approach and a designated pathogen-free (DPF) list was published by Fishman [1]. As discussed here, it is also important to also consider pathogens that may be present in the recipient that could affect the outcome and be detrimental to the xenotransplant.

Another article by Noordergraaf et al. clearly describes the challenges of maintaining a DPF donor herd and provides invaluable information on the management and exclusion of pathogens over a period of 10 years. Recommendations from their experience emphasize the need for frequent testing and also the importance of caesarean derivation [5].

Key are specific and sensitive assays - which can exclude xenozoonotic viruses - necessary to ensure that donors are not becoming a source of infection for a recipient.

Herein, is the challenge in that many of these are not available. There is a plethora of assays utilized for pathogen detection described in the literature which have been optimized, however, none are fully validated.

Essentially, testing prior to xenotransplant should be carried out by a laboratory with the relevant expertise and validated diagnostic methods. The immediate focus, assuming all other pathogens not permitted in a DPF herd have been proven absent, should be on xenozoonotic viruses. Normally, this would involve molecular analysis for presence of nucleic acid in addition to the presence or absence of antibody.

This also raises the question of what samples should be taken and stored from the donor and recipient. Should we also consider re-testing of organs from the donor post-harvest or organs/tissues to identify any possible risk? Baseline blood and tissue samples must be taken and stored in addition to a biopsy from the proposed organ for xenotransplant.

## Is There Evidence of Transmission of Porcine Viruses in Pre-Clinical Large Animal Trials?

Most of the available evidence on viruses in addition to PERV, exists for porcine cytomegalovirus (PCMV), also known as porcine roseolovirus (PCMV/PRV). While there is no conclusive evidence that PCMV/PRV infects human cells *in vitro*, transmission of PCMV/PRV to xenotransplant recipients can have harmful effects [6, 7]. High virus loads in transplanted tissues and associated health issues have been noted, particularly in baboons receiving PCMV/PRV-positive transplants [8]. A consumptive coagulopathy was observed in pig-to baboon kidney xenotransplantation, and an association of PCMV/PRV indirectly suggested by its absence in NHPS receiving organs from PCMV/PRV free donors [2]. A possible mechanism is the observed increase of porcine tissue factor in an *in vitro* model of primary porcine aortic endothelial cells infected with PCMV/PRV, providing a potential link with PCMV/PRV [9]. The role of PCMV/PRV on graft survival was demonstrated by a recently published study in an orthotopic heart transplantation model [7]. Baboon recipients of a PCMV/PRV-positive donor heart developed elevated IL-6, TNF-α and tPA-PAI-1 complex levels pointing to an interference with the cytokine and coagulation system.

Recently, circoviruses have garnered interest in xenotransplantation due to their potential for transmission, as demonstrated in a large animal trial [10]. Although early weaning can be utilized, for full exclusion, caesarean derivation has repeatedly been proposed as the highest standard, albeit it is inconsistent for PLHV [11].

## An Approach to Prevention of Transmission of Exogenous and Endogenous Viruses in Xenotransplantation

Most infectious viral pathogens can be excluded via standard surveillance and monitoring which is mainly carried out by veterinary diagnostic laboratories, e.g., Animal Plant and Health Agency (APHA, United Kingdom). In the large animal trials, strategies such as early weaning have shown some success in improving graft survival by eliminating PCMV/PRV [6, 9]. Over 200 human patients have received pig tissues for treating various conditions, such as diabetes (islet cells), hemophilia (porcine factor VIII), burns (pig skin), and neurological diseases (neuronal cells) [12]. Most of these trials did only use a low number of pig cells and/or a short duration of *ex vivo* perfusion, therefore, microbiological safety could not be tested reliably. A lack of appropriate microbiological tests did add to the challenge.

The first transplantation of a large vascularized organ into a human occurred in January 2022, involving a heart from a genetically modified pig [13]. Despite extensive genetic modifications and testing, PCMV/PRV transmission was missed, and may have contributed to the course of the patient. Recent pig kidney transplants in brain-dead individuals were terminated too early to confirm viral transmission [14, 15].

Single and double stranded DNA and RNA viruses such as member of the families of the *Adenoviridae, Anelloviridae*, *Astroviridae*, *Caliciviridae*, *Circoviridae*, *Parvoviridae*, *Reoviridae*, *Picornaviridae* and others are part of the pig virome [16]. Some viruses are typically found in pig livestock, such as porcine circoviruses (PCVs), African swine fever virus (ASFV), porcine parvoviruses (PPV), and pseudorabies virus (PrV) [17]. Swine influenza virus (SIV) causes a highly contagious viral infection [18]. So far, swine flu has only been described in a few patients usually with close contact, person-to-person infection has not been seen. The potential, however, was clearly demonstrated by the rapid spread of H1N1 in the human population and became a pandemic, causing 60 million cases, and 12,500 deaths in the United States (https://www.cdc.gov/flu/pandemic-resources/2009-h1n1-pandemic.html).

Prevention of these and other pig viruses is dependent on realiable methods of detection, and successful elimination strategies.

Vaccination does play a role in the elimination of some pathogens affecting pig livestock. From a theoretical perspective, different elimination strategies can be used alone or in combination: Caesarean delivery and early weaning, or treatment with antiviral drugs. The “designated pathogen free” status must be confirmed by repeated testing, and use of strict isolation to prevent new introduction. Importantly, for latent infections, confirmation of absence relies on tests measuring the host immune response (e.g. serology).

All the measures described above will also eliminate most if not all remaining bacterial, fungal and parasitic infections. As with allotransplantation, the risk of infection is related to exposure and the impact of immunesuppression. The main route of prevention is in robust screening of the donor animals.

## Post Xenotransplantation Screening of Recipients – Key Considerations

Currently, guidance by the FDA only discusses PERV testing in any detail (FDA 2016). As such, the early studies from xenotransplant recipients focused on the detection of PERV transmission [19].

In the last years numerous PCR-based and immunological assays have been developed to screen donor pigs for potentially zoonotic or xenozoonotic microorganisms. Similar methods are being used in preclinical trials and have been employed for use in patients in the case of clinical trials. Theoretically there is no need to test the recipients for microorganisms which were absent in the donor pig. However, in the case that viruses or other microorganisms were present in the donor pig at very low quantities and below the detection limit of the diagnostic assays used, additional screening of the patient at certain time point after the transplantation is advisable.

As PCR has limited coverage and cannot detect unknown pathogens, it is possible to use NGS and other sequencing technology to identify pathogens present in recipient samples. Meta-genomic NGS (mNGS) does this for entire genomes, while targeted NGS (tNGS) performs this for targeted regions of pathogen DNA or RNA. However, this is not as rapid a method, costs are high and standardization is necessary along with expertise to interpret the large data sets.

For some specific viruses such as PERVs, repeated testing as advised by the FDA (2016) is recommended even if donor animals with inactivated PERV are used [20].

In the recent pig to human cardiac xenotransplantation study, initial observations indicated the presence of PCMV/PRV and PCV in the patient [13]. This does not necessarily mean that the patient is experiencing infection, as it is well known that porcine cells are shed in to circulation and viral DNA/RNA nucleic acid can be detected as a result. Likewise, the use of cell-free preparations from samples does not guarantee that no porcine viral material is present [21]. Further investigation indicated that the xenotransplanted heart was positive for PCMV/PRV as was the donor animal [22]. Viral detection vs. replication and/or productive infection requires expert interpretation and appropriate range of diagnostics.

Fischer et al. described a protocol for combination testing for PCMV/PRV in donor animals to differentiate between active, latent and non-infected animals [23]. The further development and validation of methods such as these are paramount in moving forward in the clinic.

If the scenario does arise that a virus is detected in the patient, anti-virals are available but clinicians must be aware that porcine viruses respond differently, and certain options may not be suitable due to the high dosage required for an effect. For example, ganciclovir prophylaxis is of limited efficacy for PCMV/PRV compared to foscarnet and cidofovir [18].

At this moment in time, there are still some challenging questions. Donor animal samples should be correctly stored but it is not clear how long these should be held and if it is the responsibility of the sponsor. Baseline samples must be retained for both donor and recipient.

What should the approach be for monitoring recipients? What will give us confidence in the pre-xenotransplant diagnostic data from the donor animal? This again supports the need for validation of diagnostic assays and communication with experts in the field to assure the correct interpretation.

More clinical data is required and until this is achieved, it is important to have increased levels of recipient monitoring in the presence of high levels of immunesupporession and in the event of adverse events associated with potential infection.

## Conclusion: Recommendations for Clinical Trials for Donor and Recipient Safety

Some valuable lessons have been learned from the cardiac xenograft studies in the US and in NHP studies in terms of diagnostics for viral pathogens in donors.

To proceed with clinical investigations in Europe, there are some gaps in the knowledge that require to be addressed. Based on evidence to date, these recommendations should form a baseline for any xenotransplantation process.

1. Future xenotransplantations should use pigs free of potentially zoonotic viruses, achievable through sensitive virus detection and elimination strategies [24].
2. Validated assays must be used for xeno-specific pathogens and take into account the latency that may be associated with porcine herpesviruses.
3. Development of SOP for sampling and testing pre and post xenotransplantation should be developed to permit consistency and reproducibility of assays.
4. Training in compliance, sampling, archiving and diagnostics could also be considered to provide confidence in laboratory diagnostics.
5. Regular follow up of any recipient in a trial using diagnostic tools and the appropriate analysis and interpretation.

Despite efforts to screen donor pigs, unknown pathogen transmission cannot be entirely ruled out. In addition, diagnosing certain porcine pathogens requires complex interpretation and multiple testing reviewed by experts in this field. In this case, a risk assessment and strategy is required in the event of signs of infection in a recipient with early intervention with antivirals and increased monitoring protocols.

Thorough preparation and training of the transplantation team is crucial to ensure xenotransplantation safety [1, 12, 25].
